# Pan-Genome-Wide Investigation and Co-Expression Network Analysis of *HSP20* Gene Family in Maize

**DOI:** 10.3390/ijms252111550

**Published:** 2024-10-27

**Authors:** Hengyu Yan, Mingzhe Du, Jieyao Ding, Di Song, Weiwei Ma, Yubin Li

**Affiliations:** College of Agronomy, Qingdao Agricultural University, Qingdao 266000, China

**Keywords:** HSP20, *Zea* pan-genome, co-expression, gene family, heat stress

## Abstract

Heat shock protein 20 (HSP20) is a diverse and functionally important protein family that plays a crucial role in plants’ tolerance to various abiotic stresses. In this study, we systematically analyzed the structural and functional characteristics of the *HSP20* gene family within the *Zea* pan-genome. By identifying 56 *HSP20* pan-genes, we revealed the variation in the number of these genes across different maize inbreds or relatives. Among those 56 genes, only 31 are present in more than 52 inbreds or relatives. Further phylogenetic analysis classified these genes into four major groups (Class A, B, C, D) and explored their diversity in subcellular localization, physicochemical properties, and the terminal structures of those HSP20s. Through collinearity analysis and Ka/Ks ratio calculations, we found that most *HSP20* genes underwent purifying selection during maize domestication, although a few genes showed signs of positive selection pressure. Additionally, expression analysis showed that several *HSP20* genes were significantly upregulated under high temperatures, particularly in tassels and leaves. Co-expression network analysis revealed that *HSP20* genes were significantly enriched in GO terms related to environmental stress responses, suggesting that *HSP20* genes not only play key roles in heat stress but may also be involved in regulating various other biological processes, such as secondary metabolism and developmental processes. These findings expand our understanding of the functions of the maize HSP20 family and provide new insights for further research into maize’s response mechanisms to environmental stresses.

## 1. Introduction

With global warming and the increasingly frequent occurrence of extreme weather events, the environmental stresses that plants face throughout their lifecycle have become increasingly severe. Various abiotic stresses, including drought, high temperatures, flooding, and cold damage, have had significant negative impacts on plant growth and development. High-temperature stress, in particular, can lead to the disruption of protein homeostasis within plant cells, the malfunction of chloroplasts and mitochondria, and the excessive accumulation of reactive oxygen species (ROS). These changes can ultimately result in cellular damage or even death [1,2]. In response to these adverse conditions, plants have evolved a series of complex defense mechanisms, among which heat shock factors (HSFs) and heat shock proteins (HSPs) play crucial roles in maintaining cellular protein homeostasis [3].

HSFs are a highly conserved family of proteins, which include core domains such as the DNA-binding domain (DBD), oligomerization domain (OD), and nuclear localization signal (NLS). Some HSF proteins also contain a nuclear export signal (NES) and a C-terminal activation domain (CTD) [4]. The DBD allows HSFs to bind to heat shock elements (HSEs) in the promoters of target genes, thereby regulating the expression of these genes. Based on structural differences, HSFs are classified into three subfamilies: HSFA, B, and C [5]. The most widely studied role of HSFs is their involvement in heat tolerance, where they protect cells from heat-induced damage by activating heat shock proteins (HSPs).

HSPs were first discovered in *Drosophila melanogaster* exposed to heat stress [6]. Subsequent studies have shown that HSPs can be induced by high temperatures and other stresses, such as salinity, low temperature, drought, oxidative stress, and intense light [7,8,9,10]. During the stress response, HSPs help maintain cellular protein homeostasis in organisms by promoting proper protein folding, preventing the aggregation of misfolded proteins, assisting in the repair of damaged proteins, and participating in protein degradation [11,12,13,14]. HSPs are classified into five major families based on their molecular weight: HSP100, HSP90, HSP70, HSP60, and small HSPs (sHSPs or HSP20) [15]. Among them, HSP20s have the smallest molecular weight, typically ranging from 12 to 45 kDa [16]. The common structural feature of sHSPs is the conserved Alpha Crystallin Domain (ACD), which consists of a β-sandwich structure formed by seven to eight antiparallel β-strands [17]. The N-terminal and C-terminal regions of ACD are connected to highly variable N-terminal and C-terminal extensions, with the C-terminal region containing a conserved I/VXI/V motif [18]. In vivo, sHSPs usually form dynamic and flexible hollow spherical oligomers. During oligomer formation, HSP20 monomers interact through ACDs to form dimers, which then serve as the basic building blocks that assemble into higher-order oligomers through various modes [19,20]. HSP20s primarily function by binding to abnormal or unfolded proteins, preventing their aggregation. This binding ability of HSP20s inhibits the formation of insoluble aggregates from abnormal proteins, thereby protecting cells from the toxic damage associated with protein aggregation [21]. HSP20s do not have the ability to refold misfolded proteins. Instead, after stabilizing these unfolded or partially folded proteins, they pass them on to other molecular chaperones with refolding capabilities, such as HSP70 or HSP100, which are responsible for further protein repair and folding [22,23].

HSP20s play a crucial role in plant responses to adverse environmental conditions. Multiple studies have shown that overexpressing *HSP20* genes can significantly enhance the tolerance of transgenic plants to high temperatures and other stresses. For instance, overexpression of *HSP21* in transgenic *Arabidopsis* has been demonstrated to improve heat stress tolerance under high light conditions [24]. Transgenic *Arabidopsis* plants overexpressing *TaHSP23.9* from wheat (*Triticum aestivum*) exhibit enhanced tolerance to both heat and salt stress, while showing no significant differences compared to wild-type plants under normal conditions [25]. Heterologous expression of rice (*Oryza sativa*) *OsHSP20* in *E. coli* and *Pichia pastoris* has increased the thermotolerance and salt tolerance of these microorganisms. Continuous overexpression of *OsHSP20* in transgenic rice resulted in longer root systems and higher germination rates under heat and salt stress conditions [26]. Overexpression of the tomato mitochondrial small heat-shock protein (*MT-sHSP*) gene in tobacco led to transgenic plants exhibiting greater heat tolerance [27]. In *Arabidopsis*, overexpression of the *CaHsp25.9* gene from Pepper (*Capsicum annuum*) has been shown to enhance tolerance to heat, salt, and drought stress [28].

Maize, as an important global food crop, serves not only as a staple food but also plays a key role in the production of biofuels, animal feed, corn oil, and corn syrup, among other products. In recent year, the intensification of the greenhouse effect has led to frequent heatwaves during the maize growing season, severely limiting its yield. It is estimated that for every 1 °C increase in global average temperature, maize yield decreases by 7.4% [29]. Although research has demonstrated that the *HSP20* genes play critical roles in plant heat responses, the functions and regulatory mechanisms of *HSP20* genes in maize remain insufficiently explored.

Given maize’s sensitivity to heat stress, a systematic identification and functional analysis of maize *HSP20*s is of significant importance. In this study, we systematically identified members of the *HSP20* gene family across the *Zea* pan-genome and conducted a comprehensive analysis of gene structures, conserved motifs, phylogenetic relationships, protein structures, and gene expression patterns for those HSP20 proteins. Furthermore, we explored the potential functions of *HSP20* genes through co-expression network analysis and examined the co-expression relationships between *ZmHSP20*s and *ZmHSF*s. These findings provide not only a theoretical basis for a deeper understanding of the role of HSP20 in plant stress resistance but also valuable molecular resources for enhancing maize stress tolerance through genetic engineering.

## 2. Results

### 2.1. Pan-Genome-Wide Identification of HSP20s

To explore the HSP20 proteins within the *Zea* pan-genome, we first conducted an HMM (Hidden Markov Model) search in the maize B73 inbred line, targeting the conserved HSP20 domain (PF00011) and restricting the molecular weight of the proteins to between 10 kDa and 42 kDa. A total of 43 *HSP20* genes were identified, which is very close to the results previously reported in the literature [30], indicating the reliability of this method (Figure S1). Subsequently, we applied this method to identify *HSP20* genes across the *Zea* pan-genome, which includes 57 genomes from 55 maize inbreds or relatives, and discovered 56 *HSP20* pan-genes (Table 1), representing a total of 2430 *HSP20* genes across different maize inbreds or relatives (Tables S1 and S2). Among different maize inbreds and relatives, the number of *HSP20* genes ranges from 33 to 49. Specifically, there are 11 inbred lines with 33 to 40 *HSP20* genes, 37 inbred lines with 40 to 45 *HSP20* genes, and 7 inbred lines with 45 to 49 *HSP20* genes (Table S1). We then analyzed the physicochemical properties and subcellular localization characteristics of the HSP20 proteins. The amino acid sequence lengths of the HSP20 proteins ranged from 124 to 340 residues, with corresponding molecular weights ranging from 13.38 to 36.47 kDa. This variation in length and mass suggests that HSP20 proteins may possess different functional roles or adaptations. For instance, shorter HSP20 proteins may have more flexible structures, allowing them to respond more rapidly to environmental changes, while longer proteins may contain more domains, endowing them with specific functional characteristics. The aliphatic index of HSP20 proteins ranged from 54.78 to 93.01, with 66.07% (37/56) of the HSP20 proteins having an aliphatic index higher than 70. A higher aliphatic index is generally associated with increased thermal stability of proteins. Based on this indicator, we hypothesize that most HSP20 family proteins exhibit high stability under high-temperature conditions. The instability index of the HSP20 proteins ranged from 27.25 to 74.48, with 69.64% (39/56) of the HSP20 proteins having an instability index above 40, suggesting that these HSP20 proteins may be more prone to degradation and may have shorter half-lives within the cell. Among the 56 HSP20 proteins, 55 had GRAVY (Grand Average of Hydropathicity) values that were negative (−0.81 to 0), indicating strong hydrophilicity. Subcellular localization predictions indicated that HSP20 proteins are primarily localized in the cytoplasm and chloroplasts, but some are also found in mitochondria or other organelles, suggesting that HSP20 proteins may function in various cellular environments (Table 1).

**Figure 1:**
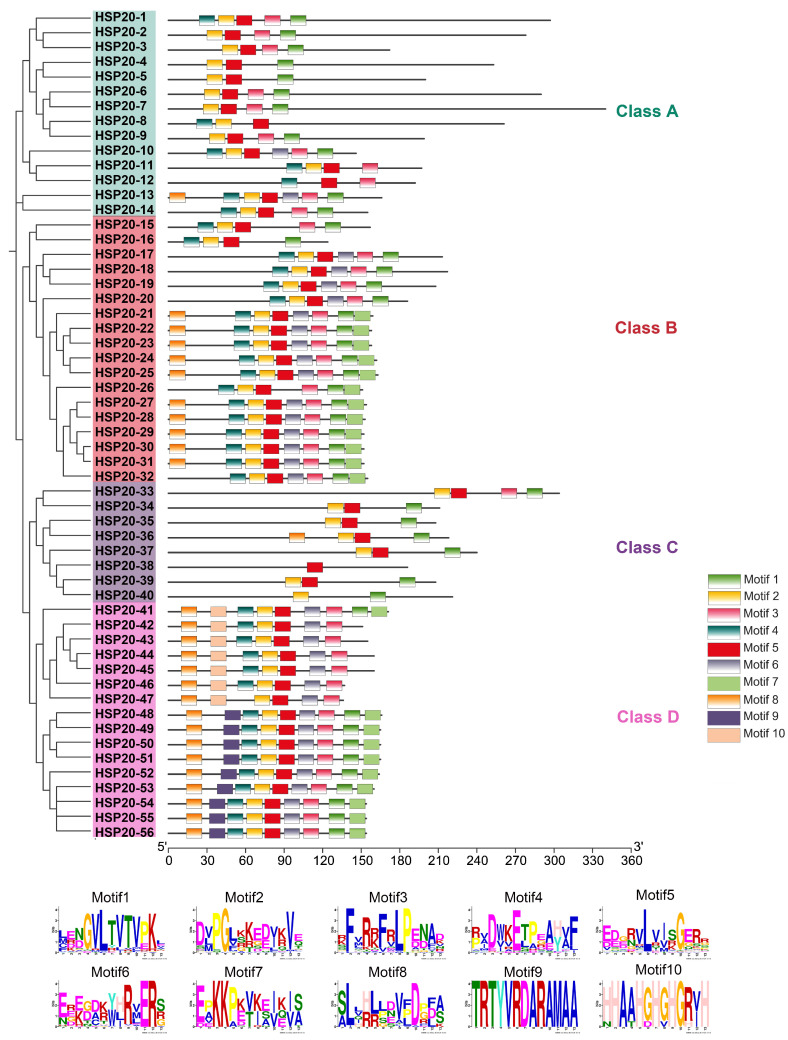
Phylogenetic analysis and conserved motif distribution of *HSP20* genes in the *Zea* pan-genome. The left panel shows a phylogenetic tree constructed based on the full-length protein sequences of the *HSP20* genes, which are categorized into four main clades: Class A (green), Class B (red), Class C (purple), and Class D (pink). The right panel displays the distribution of 10 conserved motifs, identified using the MEME tool, across these proteins. Each motif is represented by a differently colored box, with the motif numbers (Motif 1 to Motif 10) indicated. The bottom section includes the sequence logos for each of these motifs, illustrating their conserved amino acid patterns.

We conducted a phylogenetic analysis of the *HSP20* pan-genes, categorizing them into four clades: Class A, B, C, and D. Class A includes 14 proteins with diverse lengths ranging from 146 to 340 amino acids. These proteins have relatively simple conserved domains, primarily consisting of Motif 1, 2, 5, and 6 (Figure 1). Class B contains 18 proteins, most of which are approximately 160 amino acids in length, and their conserved domain composition is more complex, encompassing Motif 1, 2, 3, 4, and 5 (Figure 1). Class C consists of 8 proteins with simpler conserved domains, mainly including Motif 1, 2, 5 (Figure 1). Class D comprises 16 proteins, with lengths ranging from 136 to 171 amino acids. The diversity of conserved domains in Class D is similar to that in Class B, covering Motif 1, 2, 3, 4, 5, 6, and 8 (Figure 1). When comparing the different classes, Motif 1, 2, and 5 are almost distributed across all classes. Motif 6, 7, and 8 are predominantly found in Class B and D, whereas Motif 9 is primarily observed in Class D, potentially associated with specific functions. Overall, Class C has the simplest conserved domains, while Class B and D exhibit the most complex and diverse structures, with Class A falling in between. The distribution of these conserved domains is likely closely related to the functional specificity of HSP20 proteins within the different clades (Figure 1).

### 2.2. Presence and Absence of HSP20s in 55 Maize Inbreds or Relatives

**Figure 2:**
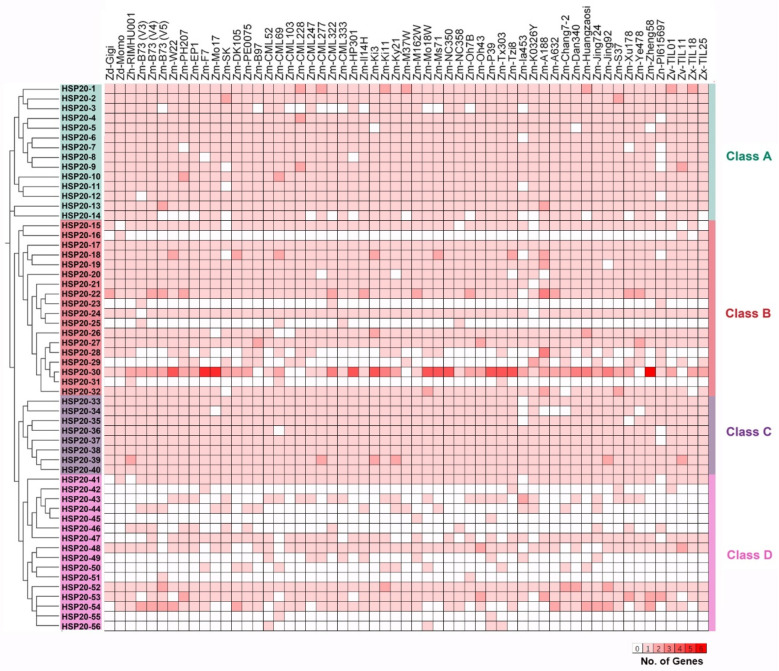
Absence and presence of *HSP20* genes across 55 maize inbred varieties. The vertical axis lists the *HSP20* genes, categorized into Class A, Class B, Class C, and Class D, each indicated by a different background color. The horizontal axis represents the names of the 55 maize inbred lines. The heatmap color intensity indicates the number of specific *HSP20* genes identified in each maize inbred line or its relatives. In certain inbred lines, a single pan-gene may correspond to multiple genes. Darker red shades represent a higher count of corresponding genes in that particular line.

We conducted a comparative analysis of the distribution of *HSP20* genes across different maize inbreds or relatives. Among the 55 maize inbreds or relatives, 28 *HSP20* genes were classified as core genes, being present in at least 95% of the pan-genome inbred lines (i.e., more than 52 lines), indicating a high level of conservation (Figure 2, Table S1). Ten of twenty-eight core genes were present in all inbred lines, including *HSP20-1*, *HSP20-2*, *HSP20-10*, *HSP20-13*, *HSP20-17*, *HSP20-27*, *HSP20-38*, *HSP20-39*, *HSP20-40*, and *HSP20-52* (Table S1). The remaining 28 *HSP20* genes were classified as dispensable genes, as they were found in fewer than 52 inbred lines, demonstrating greater variability. Notably, *HSP20-45*, *HSP20-51*, and *HSP20-55* were detected in only two inbred lines, with both *HSP20-45* and *HSP20-55* appearing in the Zm-P39 inbred line. The proportion of core genes varied significantly across different classes. In Class A and Class C, the majority of genes were core genes, with proportions of 11 out of 14 and 7 out of 8, respectively. In Class B, half of the genes (9 out of 18) were core genes, while the remaining 9 were classified as dispensable. In contrast, in Class D, only 1 out of 16 genes was classified as a core gene (Figure 2, Table S1).

### 2.3. ACD Domain in the Zea Pan-Genome

**Figure 3:**
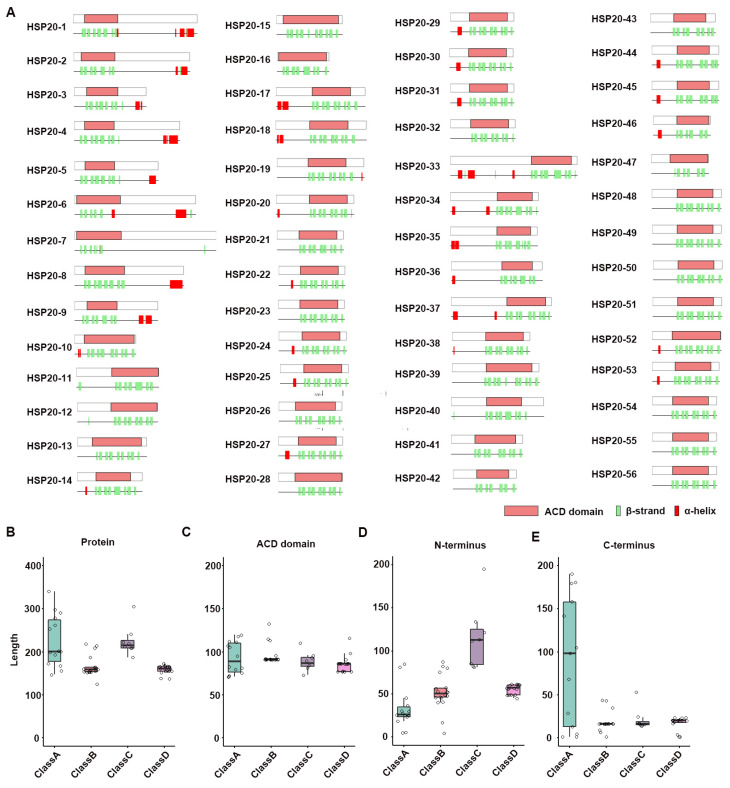
Comparative analysis of secondary structures and ACD domains in HSP20 proteins. (**A**) Diagram illustrating the alignment of secondary structures and ACD domains across various HSP20 proteins. Each gene is represented by two sections: the upper section indicates the relative position of the ACD domain within the protein, while the lower section shows the relative positions of β-strands and α-helices within the protein’s secondary structure. (**B**–**E**) Box plots displaying the distribution of lengths among different classes of HSP20 proteins: (**B**) Total protein length, (**C**) ACD domain length, (**D**) N-terminus length, and (**E**) C-terminus length.

The ACD is the core structural region of the sHSP family, and its function has been extensively studied [31]. A critical component of the ACD domain is the β-strand. We conducted a statistical analysis of the number of β-strands in the ACD domains of 56 HSP20 proteins and found that the number ranged from 5 to 10. In total, 91.07% of the ACD domains contained 5 to 8 β-strands (Figure 3A, Table S3). We performed 3D structure predictions for the maize HSP20 proteins and discovered that all these proteins share a common β-strand structure, which consists of two sheets, each composed of multiple β-strands (Figures S2–S5). The length of the ACD domains ranged from 72 to 134 amino acids, with an average length of about 91 amino acids (Table S3). There were no significant differences in the average length of ACD domains across different HSP20 protein classes. Specifically, the average length of ACD domains in Class A proteins was 93.93 amino acids, ranging from 72 to 121 amino acids; Class B proteins had the longest average ACD domain length at 97.28 amino acids, ranging from 92 to 134 amino acids; Class C proteins had an average ACD domain length of 89.88 amino acids, ranging from 74 to 111 amino acids; and Class D proteins had the shortest average ACD domain length at 87.13 amino acids, ranging from 78 to 117 amino acids. Although there were significant differences in the overall length of HSP20 proteins across different classes, the ACD domain, as a critical functional region, was relatively conserved in length across all classes (Figure 3B,C).

There were significant differences in the length of the N-terminal regions of HSP20 proteins across different classes (Figure 3D). In contrast, the length of the C-terminal regions of HSP20 proteins showed little variation across classes, except for Class A (Figure 3E). Previous studies have indicated that the C-terminal region of HSP20 proteins contains a conserved motif involved in the oligomerization process of heat shock proteins, which is referenced in the literature as I/V-X-I/V [18]. This motif is known as the C-terminal Anchoring Module (CAM). We analyzed the C-terminal regions of maize HSP20 proteins and found that 35 HSP20s contained the CAM, which was predominantly present in Class B and Class C proteins, partially present in Class D, and only one instance in Class A (Figure S6). In the CAM, the central amino acid, represented as X, was frequently found to be glutamine (Q) or glutamate (E) (Figure S6). Overall, the diversity in the terminal regions may contribute to the functional diversification of the HSP20 family, as these regions are typically involved in interactions between HSP20 proteins and their subcellular localization.

### 2.4. Chromosomal Localization, Collinearity Analysis and Selective Pressure Analysis of the HSP20 Genes in Zea Pan-Genome

**Figure 4:**
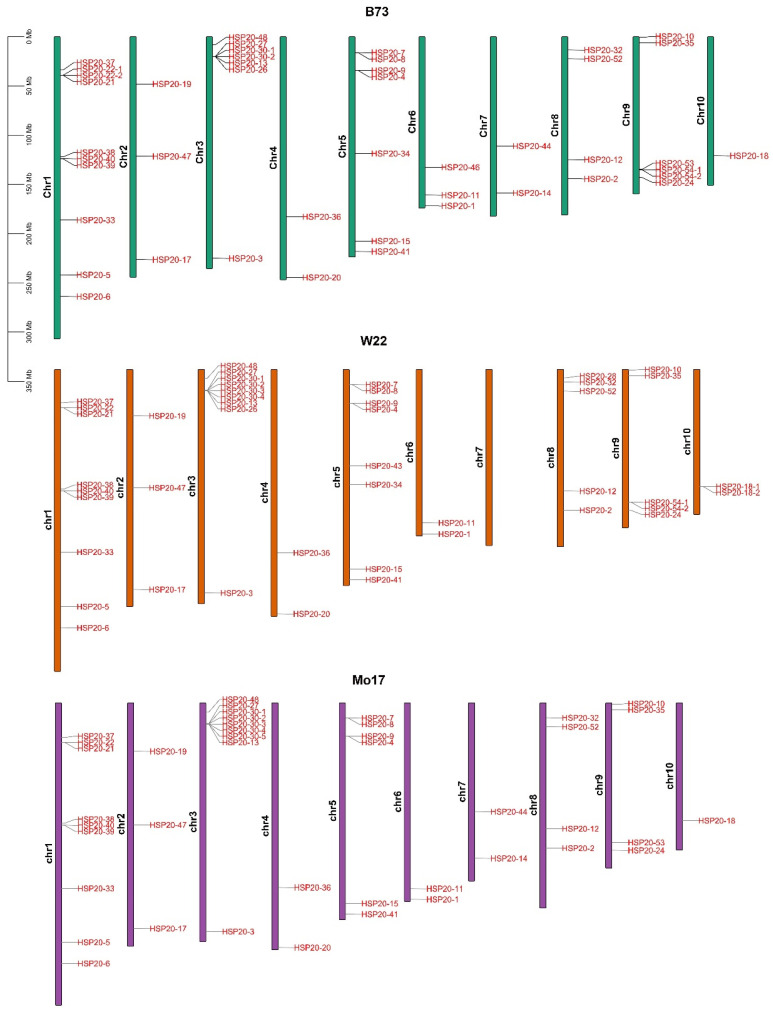
Distribution of *HSP20* genes on the chromosomes of maize inbred lines B73, W22, and Mo17. The figure illustrates the chromosomal localization of *HSP20* gene family members in three maize inbred lines. Chromosomes of different inbred lines are represented by different colors: B73 in green, W22 in orange, and Mo17 in purple. The red-labeled genes indicate the specific chromosomal positions of each *HSP20* gene.

To gain a better understanding of the chromosomal distribution of *HSP20* genes in maize, we mapped the distribution of these genes across the genomes of three maize inbred lines: B73, Mo17, and W22. The results showed that the distribution of *HSP20* genes on the chromosomes of these three inbred lines is quite dynamic (Figure 4). There is a certain similarity in the distribution patterns among the three inbred lines. First, the number of *HSP20* genes varies across different chromosomes, with chromosomes 1, 3, and 5 harboring the highest number of *HSP20* genes, while other chromosomes have relatively fewer *HSP20* genes. Second, the distribution of *HSP20* genes on individual chromosomes is also uneven, with some *HSP20* genes appearing in clusters, particularly on chromosomes 1, 3, and 9 (Figure 4).

Gene families are typically formed through gene duplication events, including tandem duplication, segmental duplication, and whole-genome duplication. We conducted a collinearity analysis to understand the formation process of the *HSP20* gene family. The results indicated that in the three maize inbred lines, the *HSP20* gene family was primarily formed through segmental and tandem duplications (Table 2). All of the duplicated *HSP20* gene pairs had Ka/Ks (Ka: non-synonymous substitutions, Ks: synonymous substitutions) ratios less than 1, suggesting that these genes have undergone purifying selection during gene duplication (Table 2). Additionally, there is some similarity in the duplication types of the same gene pairs across different inbred lines. For example, *HSP20-4:HSP20-5*, *HSP20-11:HSP20-12*, *HSP20-18:HSP20-19*, and *HSP20-35:HSP20-36* are all segmental duplications, while *HSP20-30-1:HSP20-30-2* are tandem duplications (Table 2).

**Figure 5:**
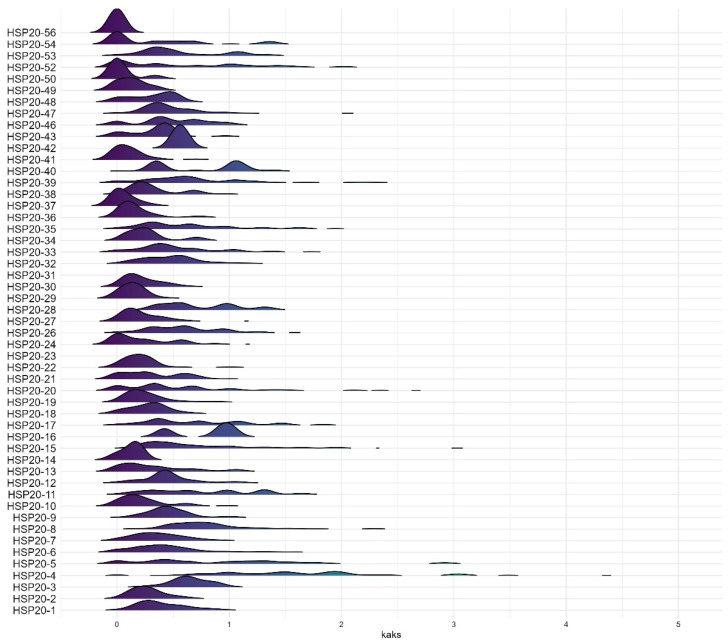
Distribution of Ka/Ks ratios of the *HSP20* gene family across different maize inbred lines in the pan-genome. This figure shows the distribution of Ka/Ks ratios for various *HSP20* genes across different maize inbred lines. The *y*-axis lists the *HSP20* genes, while the *x*-axis represents the Ka/Ks ratio values. The color gradient in the density plot, ranging from purple to yellow, indicates the magnitude of the Ka/Ks values, with darker colors representing lower ratios and lighter colors representing higher ratios. The height of the peaks indicates the frequency of occurrence of that Ka/Ks value.

**Figure 6:**
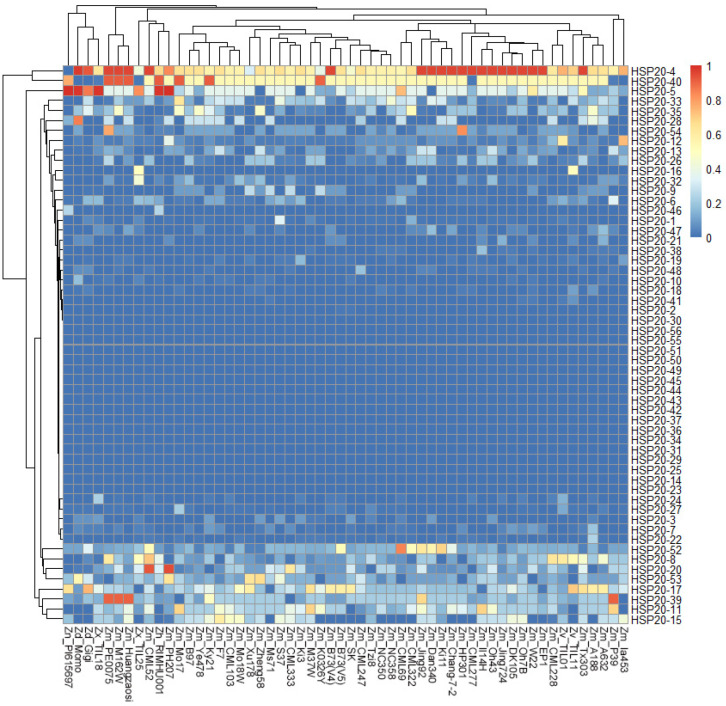
Heatmap of the frequency of occurrence of different maize varieties at each *HSP20* gene with Ka/Ks ratio > 1. The heatmap shows the frequency distribution of Ka/Ks values greater than 1 associated with specific *HSP20* genes in each maize variety. The rows represent different *HSP20* genes, while the columns represent different maize varieties. The color scale from blue to red indicates the frequency, with blue representing lower frequencies and red representing higher frequencies. The clustering of rows and columns reflects patterns of similarity in the occurrence of specific maize varieties across the *HSP20* genes.

To further investigate the selective pressures on *HSP20* genes in different maize varieties, we calculated the Ka/Ks values for each *HSP20* gene in the *Zea* pan-genome (Figure 5). The results showed that the Ka/Ks values for most *HSP20* genes ranged between 0 and 1, indicating that these genes have primarily undergone purifying selection during maize domestication (Figure 5). For instance, genes such as *HSP20-56*, *HSP20-54*, *HSP20-52*, *HSP20-41*, *HSP20-37*, *HSP20-36*, *HSP20-30*, and *HSP20-29* showed significant peaks at Ka/Ks values close to 0, suggesting that these genes have remained highly conserved across most maize varieties and have not been subjected to significant positive selection. However, some *HSP20* genes, such as *HSP20-4*, *HSP20-5*, *HSP20-11*, *HSP20-17*, and *HSP20-40*, exhibited Ka/Ks values greater than 1, potentially indicating positive selection in certain maize inbred lines (Figure 5). Further analysis of the frequency of Ka/Ks values greater than 1 for specific *HSP20* genes in different maize varieties revealed that *HSP20-4*, *HSP20-40*, and *HSP20-5* frequently exhibited Ka/Ks values greater than 1 across most maize varieties, suggesting that these genes may have been under strong positive selection pressure during maize evolution. In contrast, *HSP20-33*, *HSP20-35*, *HSP20-54*, *HSP20-52*, *HSP20-8*, *HSP20-17*, *HSP20-39,* and *HSP20-11* appeared to have experienced strong positive selection pressure only in certain maize varieties (Figure 6). In core genes, not all Ka/Ks values are less than 1. Although the frequency of Ka/Ks values greater than 1 is relatively low for most genes, *HSP20-4*, *HSP20-5*, and *HSP20-40* show a higher frequency of Ka/Ks values greater than 1 in many inbred lines, suggesting that these genes may have undergone strong positive selection pressure during maize domestication, possibly related to adaptation to specific environmental conditions or functional optimization. In contrast, *HSP20-11*, *HSP20-17*, *HSP20-39*, and *HSP20-52* exhibit a higher frequency of Ka/Ks values greater than 1 only in certain inbred lines, indicating that these genes may have experienced positive selection specific to those lines, potentially due to unique environmental adaptations or functional requirements (Figure 6).

### 2.5. Expression Analysis of Maize HSP20 Genes After Heat Stress Treatment

**Figure 7:**
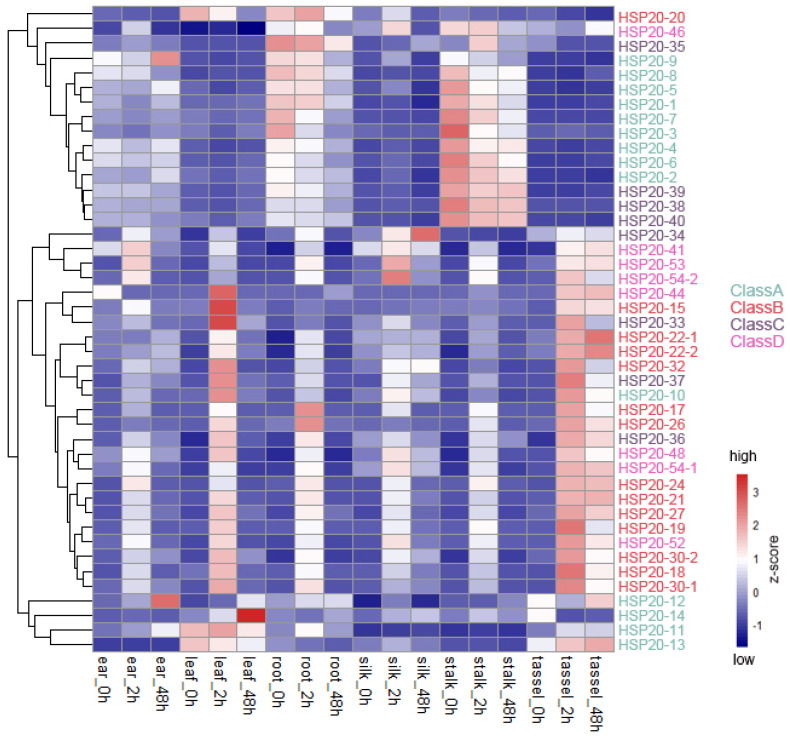
Expression of the Maize *HSP20* Genes After Heat Stress Treatment. The heatmap illustrates the expression profiles of the *HSP20* gene family in different maize (B73) tissues under heat stress treatment. The horizontal axis represents various tissues and time points, including leaves, roots, ears, silks, stalks, and tassels at 0 h, 2 h, and 48 h after heat stress treatment. The vertical axis lists the different *HSP20* genes. The color gradient from blue to red indicates the Z-score of gene expression, ranging from low (blue) to high (red) expression levels.

The *HSP20* gene family plays a crucial role in plant adaptation to heat stress. Typically, under high-temperature conditions, the expression levels of *HSP20* genes increase significantly, thereby enhancing plant heat tolerance. We explored the changes in gene expression of *HSP20* genes following heat stress treatment and observed an upregulation trend in multiple *HSP20* genes after exposure to high temperatures. This included the majority of Class B and Class D genes, as well as a few Class C and Class A genes. This upregulation was observed in various maize tissues, such as ear, leave, root, silk, stem, and tassel, with a more pronounced increase after 2 h of treatment compared to 48 h. Furthermore, the upregulation was more evident in tassels and leaves than in other tissues (Figure 7). However, not all *HSP20* genes exhibited increased expression after heat stress treatment. For instance, the expression levels of most Class A and Class C genes did not show upregulation following heat stress. In fact, under normal conditions, these genes were relatively highly expressed in stalks and roots, and their expression levels tended to decrease to some extent after heat stress treatment (Figure 7). The significant differences in response patterns among different *HSP20* gene classes across various tissues suggest that these genes may have distinct functional roles in the plant’s response to heat stress. We also used another set of expression profile data (PRJNA396192) to validate the expression patterns of *HSP20* genes discussed in this study. By comparing transcriptome data from heat-treated leaves from a public database with the expression profile data of heat-treated leaves currently in use, we found that most of *HSP20* genes exhibited similar expression patterns after heat treatment (Figure S7).

### 2.6. Co-Expression Network Analysis of Maize HSP20

**Figure 8:**
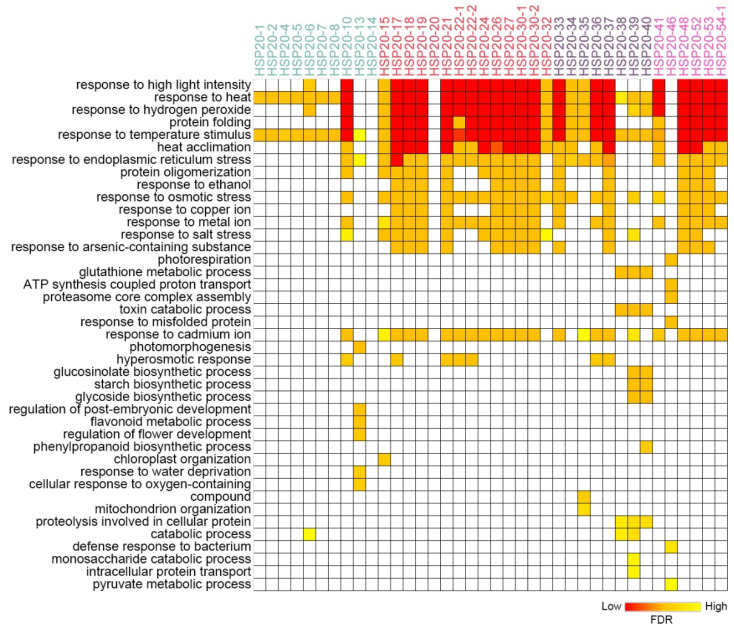
Comparative GO Enrichment Analysis for Co-expressed Genes of Maize *HSP20* Genes. This heatmap presents the GO enrichment analysis results of maize *HSP20* gene family co-expressed genes. The *x*-axis represents individual *HSP20* genes, with font colors indicating different categories. The *y*-axis lists the enriched GO terms related to various biological processes. The color gradient within the heatmap reflects the degree of enrichment, with deeper red colors indicating lower FDR values, signifying a higher level of enrichment for the corresponding GO terms.

Co-expression network analysis is an effective method for predicting gene functions. We conducted a co-expression network analysis of maize *HSP20* genes and downloaded the top 50 co-expressed genes for each *HSP20* gene from the ATTED database [32]. These gene sets were then subjected to GO (Gene Ontology) enrichment analysis. For genes without available co-expression networks, no GO enrichment analysis was performed. We found that many of the co-expressed genes with *HSP20* were significantly enriched in the following GO terms: “response to heat”, “response to high light intensity”, “response to hydrogen peroxide”, “response to temperature stimulus”, “protein folding”, “response to endoplasmic reticulum stress”, and “response to salt stress” (Figure 8, Table S4). These findings are consistent with our expectations regarding the potential role of *HSP20* genes in plant responses to environmental stress, further supporting the importance of *HSP20* in coping with environmental stress and maintaining cellular homeostasis. Comparatively, the co-expressed genes of Class B, C, and D showed higher enrichment in these GO terms than those of Class A (Figure 8). Additionally, we identified enrichment in other types of GO terms, such as “glucosinolate biosynthetic process”, “glycoside biosynthetic process”, and “defense response to bacterium” among the co-expressed genes of *HSP20-39* and *HSP20-40*, and “photomorphogenesis” and “regulation of flower development” in the co-expression network of *HSP20-13* (Figure 8). This suggests that, in addition to playing a role in the response to environmental stress, *HSP20* genes may also be involved in regulating various other biological processes. The enrichment of these additional GO terms hints at the broader functions of *HSP20* genes in secondary metabolism, plant defense mechanisms, and developmental regulation.

**Figure 9:**
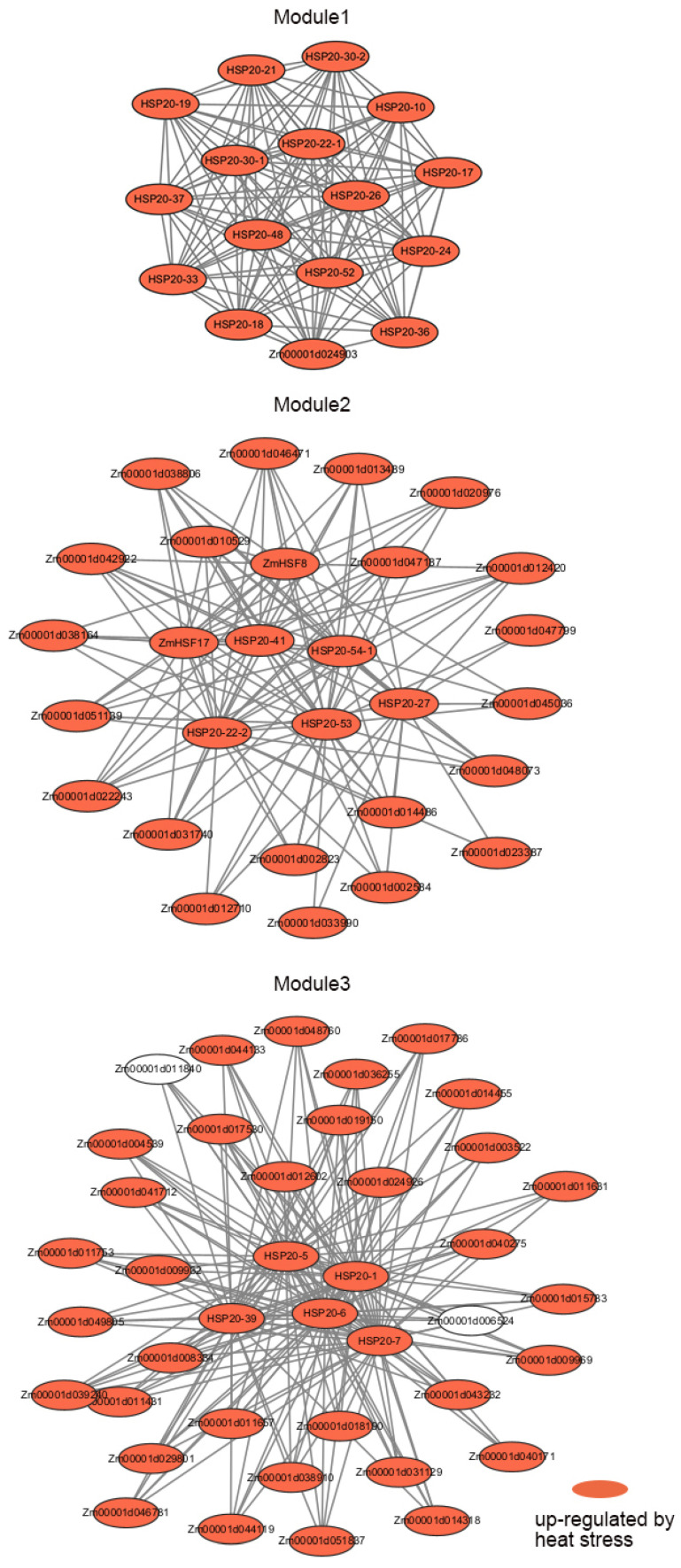
Co-expression modules of maize *HSP20* and *HSF* genes. This figure shows the co-expression modules derived from maize *HSP20* and *HSF* co-expression network. Each node represents a gene, and the connections between nodes indicate co-expression relationships. The red nodes represent genes that are up-regulated under heat stress conditions.

Given the similarity in GO function enrichment among the co-expressed gene sets of several *HSP20* genes, we hypothesized that *HSP20s* might form a co-expressed functional module. Therefore, we integrated all co-expressed genes of *HSP20s* into a single co-expression network to explore the presence of key co-expression modules. Since previous studies have reported a close relationship between *HSF* and *HSP* genes [33], we also integrated the co-expressed genes of *HSF*, ultimately forming a combined *HSP20* and *HSF* co-expression network. We then used Cytoscape’s Cfinder tool to identify key modules, ultimately identifying three significantly enriched modules. Module 1 mainly consisted of *HSP20* family members, which were highly interconnected. This module included *HSP20* genes such as *HSP20-30-2*, *HSP20-21*, *HSP20-19*, *HSP20-17*, and *HSP20-48*, as well as one non-*HSP20* family gene, *Zm00001d024903*, which belongs to the *HSP90* family (Figure 9). GO enrichment analysis revealed that the genes in Module 1 were significantly enriched in biological processes such as “response to heat”, “response to hydrogen peroxide”, “protein oligomerization”, “response to reactive oxygen species”, and “protein folding”, indicating that these genes play a critical role in responding to heat stress and oxidative stress (Figure S8). Module 2 exhibited a more complex network structure, with core genes including several *HSP20* genes (*HSP20-41*, *HSP20-54-1*, *HSP20-53*, *HSP20-22-2*, *HSP20-27*) as well as members of the HSF family (*ZmHSF8* and *ZmHSF17*) (Figure 9). Under heat stress conditions, HSFs regulate stress responses by controlling the expression of *HSP20* genes. GO analysis indicated that, in addition to the GO terms enriched in Module 1, Module 2 was also significantly enriched in terms related to protein folding, such as “chaperone-mediated protein folding requiring cofactor”, “cellular response to topologically incorrect protein”, and “protein refolding”, suggesting an important role for this module in maintaining cellular protein homeostasis (Figure S8). Module 3 displayed the broadest network, involving multiple *HSP20* genes and other genes. The core *HSP20* genes in this module, such as *HSP20-1*, *HSP20-6*, *HSP20-7,* and *HSP20-39*, exhibited extensive connectivity, indicating their importance in stress responses (Figure 9).

## 3. Discussion

The *HSP20* family is the largest within the HSP superfamily. In the *Zea* pan-genome, the *HSP20* gene family exhibits significant variation in number. Through HMM search and strict molecular weight filtering, we identified 56 *HSP20* pan-genes in the *Zea* pan-genome, representing 2366 distinct *HSP20* genes. The number of these genes in different maize inbreds or relatives ranges from 31 to 44, indicating that gene amplification or loss events frequently occur in the maize genome. There are also significant differences in the number of *HSP20* genes across different species. For example, *Arabidopsis* (19, 119.1 Mb) [31], rice (39, 385.7 Mb) [34], tomato (42, 827.4 Mb) [35], potato (48, 705.8 Mb) [36], soybean (51, 978.4 Mb) [37], upland cotton (94, 2.3 Gb) [38], and bread wheat (117, 14.6 Gb) [39] have varying numbers of *HSP20* genes. Genome size data were sourced from the NCBI Genome database. The relatively few *HSP20* genes in *Arabidopsis* are somewhat correlated with its smaller genome size, while the higher numbers in upland cotton and bread wheat are associated with their larger genomes [36]. However, this correlation is not absolute. For instance, rice has a similar number of *HSP20* genes to tomato and maize, despite having a genome that is roughly half the size of tomato and one-sixth the size of maize. This phenomenon may be due to independent gene duplication events in different species [40]. In this study, we found that while the three maize inbred lines share some duplicated gene pairs, they also possess different duplicated gene pairs. This suggests that some of these gene duplication events may have occurred independently in different genetic backgrounds during the formation of specific inbred lines, leading to varying degrees of *HSP20* gene amplification in specific inbred lines. Additionally, presence/absence analysis showed that 25 out of the 56 *HSP20* genes are not present in all inbred lines, with most of the Class D *HSP20* genes being classified as dispensable. This indicates that *HSP20* genes may have undergone different levels of gene amplification or loss in different genotypes.

The functions of the *HSP20* family proteins are not limited to heat stress response. Through co-expression network analysis, this study reveals that HSP20 proteins may play roles in various other biological processes. The GO enrichment analysis of *HSP20* co-expressed genes shows significant enrichment in multiple GO terms related to environmental stress responses, such as “response to high light intensity”, “response to hydrogen peroxide”, “response to temperature stimulus”, “response to heat”, and “response to salt stress”. This indicates that HSP20 proteins are not only crucial in heat stress response but also play key roles in responding to high light, oxidative stress, salt stress, and other stress conditions. In fact, several studies have confirmed the involvement of HSP20 in these biological processes. For example, under high light conditions, the transcription level of the *HSP17.4CI* gene in *Arabidopsis* increases significantly, rising 10 and 14 fold after 6 and 12 h treatment, respectively [41]. Tomato *HSP21* can protect photosystem II from oxidative stress damage induced by temperature [42]. Overexpression of *Lilium davidii LimHSP16.45* enhances cell viability in *Arabidopsis* under various abiotic stress conditions, including high temperature, high salt, and oxidative stress [43]. Moreover, *HSP20* may also be involved in key biological processes such as secondary metabolism and developmental regulation. For instance, the co-expressed genes of *HSP20-39* and *HSP20-40* are significantly enriched in secondary metabolism processes, such as “glucosinolate biosynthetic process” and “glycoside biosynthetic process”. The co-expressed genes of *HSP20-13* are enriched in “regulation of flower development” and “photomorphogenesis”. Although there is currently no direct experimental evidence showing that *HSP20* is involved in secondary metabolism and flowering regulation, studies have shown that other types of HSP proteins participate in these processes. For example, the glucosinolate metabolism mutant *TU8* in *Arabidopsis* is sensitive to high temperatures, primarily due to defective expression of *HSP90* in the mutant [44]. Additionally, exogenous application of isothiocyanates to *Arabidopsis* enhances heat tolerance and induces the expression of *HSP70* genes [45], highlighting the close relationship between HSP proteins and plant secondary metabolism. Furthermore, localized reduction of *HSP90* expression in shoot apices prevents flower formation [46]. A mutation in *HSP70-16* disrupts the normal development and function of floral organs, such as stamens and pistils, significantly reducing fertility under both normal and mild heat stress conditions [47]. However, despite the fact that HSP70 and HSP90, which belong to the same HSP superfamily, have been shown to participate in biological processes related to secondary metabolism and developmental regulation, the specific mechanisms by which HSP20 proteins contribute to these processes remain unclear. Therefore, future research is urgently needed to uncover the detailed roles of HSP20 in these processes.

Co-expression modules are crucial for uncovering potential regulatory mechanisms [48]. Genes within the same module may be co-regulated by the same transcription factors or epigenetic modifications, providing deep insights into the regulatory networks of specific biological processes. During the heat stress response, *HSF*s play a role by activating the expression of a series of *HSP*s. This study, through co-expression network analysis, demonstrated the close co-expression relationship between *HSP20* and *HSF* in maize. The network structure within co-expression module 2 shows that multiple *HSP20* genes form a highly complex co-expression network with *HSF* genes, such as *ZmHSF8* and *ZmHSF17*. GO enrichment analysis of this module revealed its key role in maintaining cellular protein homeostasis under heat stress. For example, the genes in module 2 are significantly enriched in biological processes such as “response to heat”, “chaperone-mediated protein folding requiring cofactor”, “cellular response to topologically incorrect protein”, and “protein refolding”. *ZmHSF17*’s homolog in *Arabidopsis* is *ATHSFA2* (*AT2G26150*), a key positive regulator of heat memory in *Arabidopsis* [49,50,51]. *HSFA2* regulates a series of heat stress-related genes, including several *HSP20s* (such as *HSP18*, *HSP21*, *HSP22*), the heat stress-associated 32-KD protein (*HSA32*), ascorbate peroxidase 2 (*APX2*), and fructose-bisphosphate aldolase 6 (*FBA6*) [24,52,53,54]. The expression of these downstream genes is crucial for maintaining plant heat memory. The co-expression relationship between *ZmHSF17* and multiple *HSP20* genes in our module suggests that a similar molecular mechanism may exist in maize, but further experimental evidence is needed to confirm this.

## 4. Materials and Methods

### 4.1. Identification of HSP20s in the Zea Pan-Genome

The protein sequence files for the *Zea* pan-genome were downloaded from MaizeGDB (https://download.maizegdb.org/Pan-genes/Pan-Zea/, accessed on 16 June 2024) [55]. The pan-genome includes 57 genome datasets from 55 maize inbreds or relatives, with Zm-B73 (V3), Zm-B73 (V4), and Zm-B73 (V5) representing different versions of the genome for the same inbred line, B73. The localized InterProScan 5 [56] software was employed to search all pan-*Zea* protein sequences for the presence of the conserved HSP20 domain (PF00011), using the default parameters. Additionally, the presence of other key HSP20 domains, such as cd06464 and cd06472 from the CDD database, and PS01031 and PS51203 from PROSITE database, was also assessed. Based on these criteria, proteins were classified as HSP20 proteins. Proteins with a molecular weight outside the 10–42 kDa range were subsequently excluded from further analysis. When a gene is present in 90% to 95% of the individuals, it is classified as part of the core genes, following the criteria proposed in previous studies [57]. Genes not included in the core genome are, by default, categorized into the dispensable genes.

### 4.2. Prediction of Protein Physicochemical Properties and Subcellular Localization

The molecular weight, aliphatic index, isoelectric point, instability index, and grand average of hydropathy (GRAVY) of HSP20 proteins were calculated using the Protein Parameter Calc program in TBtools V2024.1.11 [58]. The subcellular localization of the proteins was predicted using WoLF PSORT webserver (https://wolfpsort.hgc.jp/, accessed on 16 June 2024) [59].

### 4.3. Phylogenetic and Conserved Motif Analysis

Multiple sequence alignment of proteins of maize *HSP20* pan-genes was performed using Clustal Omega V1.2.4. Next, the aligned sequence file was automatically trimmed with TrimAl to remove spurious sequences and poorly aligned regions. Finally, a phylogenetic tree was constructed using MEGA11 [60] based on the trimmed sequence file, with default parameters. The MEME software V5.0.5 [61] was used to identify motifs in the HSP20 protein sequences. The parameters used were: “-nmotifs 10 -mod zoops -minw 6 -maxw 13 -objfun classic -markov_order 0”.

### 4.4. Prediction of Protein Secondary and Tertiary Structures

The secondary structure of the HSP20 protein sequences was predicted using the JPred4 tool (https://www.compbio.dundee.ac.uk/jpred/, accessed on 16 June 2024). The tertiary structure of the HSP20 protein sequences was predicted using ColabFold v1.5.5 (https://colab.research.google.com/github/sokrypton/ColabFold/blob/main/AlphaFold2.ipynb, accessed on 16 June 2024), a user-friendly implementation of AlphaFold2 available through Google Colab.

### 4.5. Chromosomal Localization, Gene Duplication Analysis, and Ka/Ks Calculation

Chromosomal Localization Analysis: Genome annotation files for B73, Mo17, and W22 were obtained from MaizeGDB, along with the *HSP20* gene IDs from each inbred line. Chromosomal localization was visualized using the “Gene Location Visualize from GTF/GFF” tool in TBtools.

Gene Duplication Analysis: Similar protein sequence pairs were identified across the whole genome using the BlastP tool. Gene duplication types were determined using MCScanX software by incorporating gene positional information. If the BLASTP results showed a rank difference of 1 between two genes, they were considered “tandem duplicates”. Genes located in collinear blocks were classified as “whole-genome duplications (WGD)/segmental duplications”.

Ka/Ks Analysis: Ka and Ks, as well as Ka/Ks ratios, were calculated using KaKs_Calculator V2.0. Generally, Ka/Ks = 1 is defined as a neutral mutation, Ka/Ks > 1 indicates positive selection, and Ka/Ks < 1 suggests negative (purifying) selection.

### 4.6. Gene Expression Analysis

Gene expression profile data were obtained from the PlantrnaDB (https://plantrnadb.com//zmrna/download/, accessed on 16 June 2024, PRJNA520822 and PRJNA396192). Expression levels were standardized using Z-score normalization, and clustering was performed using the Pheatmap package in R V4.4.1.

### 4.7. Co-Expression Network Analysis and GO Enrichment Analysis

The top 50 co-expressed genes for maize *HSP20* and *HSF* were downloaded from the ATTED-II database, and a co-expression network was constructed using Cytoscape. Co-expression modules were generated with the Cfinder tool in Cytoscape, utilizing default parameters. GO enrichment analysis of the HSP20 co-expressed gene set and key co-expression modules was conducted using AgriGOv2 (https://systemsbiology.cau.edu.cn/agriGOv2/index.php, accessed on 16 June 2024). The background gene set for the analysis was Maize v4 (Maize-GAMER), with the enrichment significance level set to FDR ≤ 0.05.

## 5. Conclusions

In conclusion, we have conducted a thorough analysis of the *HSP20* gene family within the *Zea* pan-genome, revealing variations in the number of *HSP20* genes among different maize inbreds or relatives. We identified 56 pan-genes, of which 28 are dispensable genes. Those genes are often overlooked in traditional gene family identification based on single genomes. The observed upregulation of some *HSP20* genes under heat stress underscores their pivotal role in enhancing maize heat tolerance. Additionally, *HSP20* genes appear to play important roles not only in heat stress response but also under various environmental conditions such as high light intensity, oxidative stress, and salt stress, as well as in processes like secondary metabolism and developmental regulation. The co-expression network analysis further highlights the close interactions between *HSP20* and *HSF* genes, indicating the formation of complex regulatory networks that contribute to cellular protein homeostasis and environmental stress responses. In summary, these findings enhance our understanding of the molecular mechanisms behind maize’s response to environmental stress and offer valuable new targets for improving stress tolerance in maize.

## Figures and Tables

**Table 1:** Identification of HSP20 Proteins from the Maize Pangenome.

Pangene	Name	Protein Length	Molecular Weight (Da)	Aliphatic Index	Isoelectric Point	Instability Index	GRAVY	Subcellular Localization
pan-zea.v2.pan00826	HSP20-1	297	31,516.61	61.45	9.19	54.37	−0.73	Cytosol
pan-zea.v2.pan12077	HSP20-2	278	30,438.69	67.88	7.00	40.45	−0.81	Cytosol
pan-zea.v2.pan26496	HSP20-3	172	18,232.62	77.97	7.71	35.52	−0.12	Mitochondrion
pan-zea.v2.pan08986	HSP20-4	253	27,068.95	67.83	8.87	41.74	−0.61	Cytosol
pan-zea.v2.pan14219	HSP20-5	200	21,799.43	69.65	9.94	55.15	−0.58	Golgi apparatus
pan-zea.v2.pan04337	HSP20-6	290	31,742.52	62.69	6.77	57.02	−0.74	Golgi apparatus
pan-zea.v2.pan16262	HSP20-7	340	36,473.84	63.85	9.57	70.67	−0.69	Cytosol
pan-zea.v2.pan20944	HSP20-8	261	28,679.94	69.20	9.66	40.72	−0.81	Chloroplast
pan-zea.v2.pan02205	HSP20-9	199	21,548.15	82.81	6.71	42.01	−0.34	Cytosol
pan-zea.v2.pan02580	HSP20-10	146	15,845.89	81.44	8.06	40.06	−0.26	Peroxisome
pan-zea.v2.pan10469	HSP20-11	197	21,876.25	69.49	4.75	42.86	−0.32	Chloroplast
pan-zea.v2.pan18435	HSP20-12	192	21,341.64	68.70	4.82	59.88	−0.35	Chloroplast
pan-zea.v2.pan01914	HSP20-13	166	17,382.48	93.01	5.02	53.42	0.13	Cytosol
pan-zea.v2.pan25585	HSP20-14	155	17,160.18	77.48	6.59	41.58	−0.35	Cytosol
pan-zea.v2.pan23877	HSP20-15	157	16,686.51	54.78	7.78	46.16	−0.61	Cytosol
pan-zea.v2.pan49059	HSP20-16	124	13,387.90	62.18	6.41	39.87	−0.55	Cytosol
pan-zea.v2.pan05749	HSP20-17	213	22,736.75	90.66	6.34	46.02	−0.03	Extracellular
pan-zea.v2.pan01221	HSP20-18	217	23,437.10	81.01	6.08	44.48	−0.41	Extracellular
pan-zea.v2.pan05142	HSP20-19	208	22,831.56	87.26	6.00	49.55	−0.34	Chloroplast
pan-zea.v2.pan22794	HSP20-20	186	20,163.50	76.13	6.85	53.95	−0.54	Chloroplast
pan-zea.v2.pan02999	HSP20-21	159	17,879.91	69.87	6.86	52.94	−0.67	Cytosol
pan-zea.v2.pan00785	HSP20-22	158	17,785.70	65.38	5.55	58.00	−0.67	Cytosol
pan-zea.v2.pan60829	HSP20-23	158	17,758.67	65.38	5.55	59.75	−0.65	Cytosol
pan-zea.v2.pan12992	HSP20-24	162	17,888.68	61.98	5.53	56.43	−0.61	Cytosol
pan-zea.v2.pan52633	HSP20-25	163	18,021.89	63.99	5.53	52.39	−0.56	Cytosol
pan-zea.v2.pan15310	HSP20-26	151	16,903.81	74.24	6.19	48.79	−0.62	Cytosol
pan-zea.v2.pan01326	HSP20-27	154	17,058.04	77.79	6.77	51.44	−0.45	Cytosol
pan-zea.v2.pan30055	HSP20-28	153	17,216.27	83.99	5.71	52.87	−0.42	Cytosol
pan-zea.v2.pan35919	HSP20-29	152	17,131.14	73.03	5.81	51.53	−0.60	Cytosol
pan-zea.v2.pan00137	HSP20-30	152	17,223.25	69.80	5.81	50.97	−0.65	Cytosol
pan-zea.v2.pan49535	HSP20-31	152	17,253.34	69.80	5.81	53.86	−0.63	Cytosol
pan-zea.v2.pan11491	HSP20-32	155	17,338.34	77.23	5.82	60.63	−0.55	Cytosol
pan-zea.v2.pan03732	HSP20-33	304	33,110.86	74.51	9.75	53.27	−0.50	Chloroplast
pan-zea.v2.pan09257	HSP20-34	211	22,818.40	88.39	6.86	36.86	−0.39	Chloroplast
pan-zea.v2.pan12441	HSP20-35	208	22,276.25	89.57	5.50	27.25	−0.27	Cytosol
pan-zea.v2.pan20814	HSP20-36	218	23,815.70	86.79	6.47	42.75	−0.47	Chloroplast
pan-zea.v2.pan13949	HSP20-37	240	26,377.65	73.92	7.86	52.63	−0.54	Chloroplast
pan-zea.v2.pan03513	HSP20-38	186	20,301.00	82.26	8.89	47.76	−0.35	Chloroplast
pan-zea.v2.pan00862	HSP20-39	208	22,619.46	82.45	9.37	74.48	−0.43	Chloroplast
pan-zea.v2.pan03519	HSP20-40	221	23,927.02	77.24	9.30	61.07	−0.47	Chloroplast
pan-zea.v2.pan23388	HSP20-41	171	18,347.54	80.94	6.60	36.72	−0.45	Cytosol
pan-zea.v2.pan85222	HSP20-42	151	16,794.63	68.41	6.17	46.34	−0.61	Cytosol
pan-zea.v2.pan30437	HSP20-43	155	17,020.06	86.13	6.05	34.40	−0.33	Cytosol
pan-zea.v2.pan38397	HSP20-44	160	17,455.56	81.06	6.16	39.87	−0.37	Cytosol
pan-zea.v2.pan75273	HSP20-45	160	17,455.56	81.06	6.16	39.87	−0.37	Cytosol
pan-zea.v2.pan44086	HSP20-46	137	14,983.57	77.52	5.72	42.60	−0.53	Cytosol
pan-zea.v2.pan27498	HSP20-47	136	15,082.05	84.56	8.66	41.49	−0.48	Cytosol
pan-zea.v2.pan05999	HSP20-48	166	17,967.58	79.88	5.95	35.62	−0.32	Cytosol
pan-zea.v2.pan79512	HSP20-49	165	17,896.51	79.76	5.95	35.77	−0.34	Cytosol
pan-zea.v2.pan41785	HSP20-50	165	17,868.45	78.61	5.95	35.72	−0.35	Cytosol
pan-zea.v2.pan61143	HSP20-51	165	17,868.45	78.61	5.95	35.72	−0.35	Cytosol
pan-zea.v2.pan01495	HSP20-52	164	17,799.24	79.63	5.33	37.95	−0.35	Nucleus
pan-zea.v2.pan19046	HSP20-53	160	17,444.91	84.13	6.17	34.60	−0.32	Nucleus
pan-zea.v2.pan25972	HSP20-54	154	17,046.54	83.57	7.84	35.01	−0.36	Cytosol
pan-zea.v2.pan64728	HSP20-55	154	17,046.54	83.57	7.84	35.01	−0.36	Cytosol
pan-zea.v2.pan80457	HSP20-56	154	17,046.54	83.57	7.84	35.01	−0.36	Cytosol

**Table 2:** Ka/Ks Analysis of Duplicated *HSP20* Genes in Maize.

Inbred	Duplicated Pairs	Ka	Ks	Ka/Ks	Duplication Type
B73	*HSP20-2:HSP20-3*	0.530	1.076	0.493	SD
	*HSP20-4:HSP20-5*	0.148	0.208	0.714	SD
	*HSP20-6:HSP20-7*	0.199	0.243	0.819	SD
	*HSP20-11:HSP20-12*	0.065	0.104	0.622	SD
	*HSP20-18:HSP20-19*	0.064	0.185	0.348	SD
	*HSP20-24:HSP20-22-1*	0.062	0.484	0.128	SD
	*HSP20-27:HSP20-32*	0.087	0.347	0.249	SD
	*HSP20-35:HSP20-36*	0.415	0.646	0.643	SD
	*HSP20-48:HSP20-52*	0.055	0.147	0.373	SD
	*HSP20-30-1:HSP20-30-2*	0.999			TD
	*HSP20-39:HSP20-40*	0.596	0.990	0.602	TD
Mo17	*HSP20-1:HSP20-3*	0.612	1.320	0.464	SD
	*HSP20-2:HSP20-3*	0.505	1.134	0.445	SD
	*HSP20-4:HSP20-5*	0.139	0.188	0.742	SD
	*HSP20-11:HSP20-12*	0.065	0.095	0.683	SD
	*HSP20-18:HSP20-19*	0.066	0.156	0.422	SD
	*HSP20-30-4:HSP20-32*	0.059	0.130	0.456	SD
	*HSP20-36:HSP20-35*	0.451	0.801	0.563	SD
	*HSP20-48:HSP20-52*	0.048	0.129	0.371	SD
	*HSP20-30-1:HSP20-30-2*	0.113	0.226	0.501	TD
	*HSP20-30-3:HSP20-30-4*	0.133	0.281	0.473	TD
W22	*HSP20-4:HSP20-5*	0.148	0.208	0.713	SD
	*HSP20-6:HSP20-7*	0.180	0.237	0.759	SD
	*HSP20-11:HSP20-12*	0.065	0.104	0.622	SD
	*HSP20-18-1:HSP20-19*	0.064	0.168	0.385	SD
	*HSP20-30-4:HSP20-32*	0.060	0.173	0.349	SD
	*HSP20-35:HSP20-36*	0.411	0.664	0.619	SD
	*HSP20-27:HSP20-30-1*	0.075	0.436	0.172	TD
	*HSP20-30-1:HSP20-30-2*	0.003	0.118	0.024	TD
	*HSP20-30-2:HSP20-30-3*	0.020	0.073	0.272	TD
	*HSP20-39:HSP20-40*	0.654	0.990	0.660	TD

SD: Segment Duplication; TD: Tandem Duplication.

## Data Availability

The original contributions presented in the study are included in the article/Supplementary Material, further inquiries can be directed to the corresponding authors.

## Supplementary Materials

The supporting information can be downloaded at: https://www.mdpi.com/article/10.3390/ijms252111550/s1.
