# Supplementary material for: Pan-Genome-Wide Investigation and Co-Expression Network Analysis of HSP20 Gene Family in Maize
Source: Int J Mol Sci. 2024 Oct 27;25(21):11550. doi: 10.3390/ijms252111550 (PMC11546149; doi:10.3390/ijms252111550)
Supplement: Supplementary file 1 [file ijms-25-11550-s001.zip › captions.pdf]

Figure S1: Venn Diagram Showing the Consistency Between In-House Predicted HSP20 in B73 and Smith's Prediction;
Figure S2: 3D Structure of Class A HSP20 Proteins;
Figure S3: 3D Structure of Class B HSP20 Proteins;
Figure S4: 3D Structure of Class C HSP20 Proteins;
Figure S5: 3D Structure of Class D HSP20 Proteins;
Figure S6: Multiple Sequence Alignment of the C-Terminus of the ACD Domain in Maize HSP20 Proteins, with the Red Box Highlighting the C-Terminal I/V-X-I/V Motif;
Figure S7: The expression of *HSP20* genes in two sets of heat-treated leaf transcriptome data (PRJNA520822, PRJNA396192);
Figure S8: GO enrichment result of Co-exprssion module 1, 2 and 3;
Table S1: HSP20 genes identified in 55 maize inbreds or relatives;
Table S2: HSP20 pan-genes identified from *Zea* pan-genome;
Table S3: ACD Domain and Secondary Structure Information of HSP20s;
Table S4: GO Enrichment Analysis Results of Co-expressed Genes of Maize *HSP20* Genes;
