# Supplementary figures and images for: Pan-Genome-Wide Investigation and Co-Expression Network Analysis of HSP20 Gene Family in Maize
Source: Int J Mol Sci. 2024 Oct 27;25(21):11550. doi: 10.3390/ijms252111550 (PMC11546149; doi:10.3390/ijms252111550)

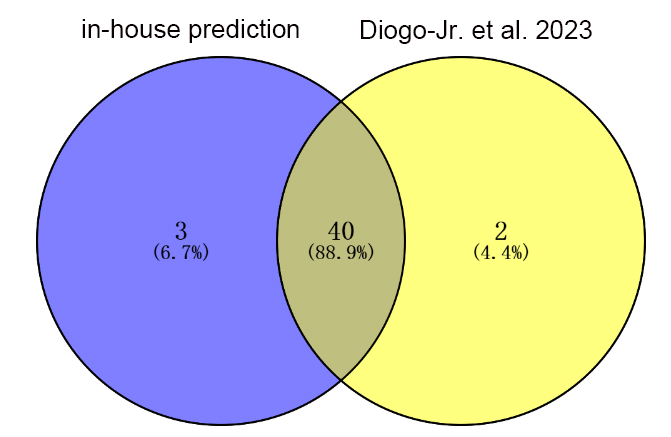

Supplement: Supplementary file 1 [file ijms-25-11550-s001.zip › FigureS1.jpg]

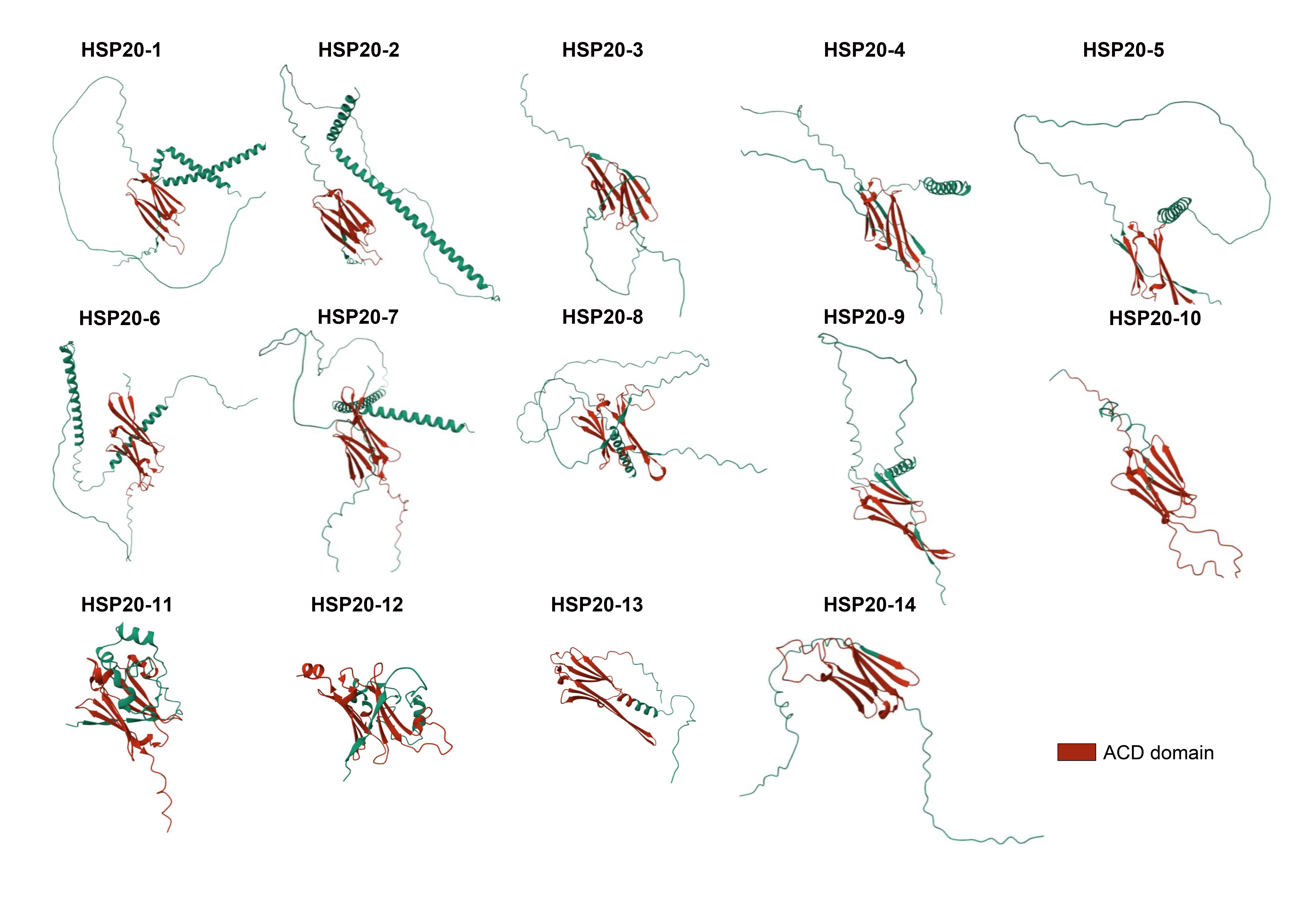

Supplement: Supplementary file 1 [file ijms-25-11550-s001.zip › FigureS2.jpg]

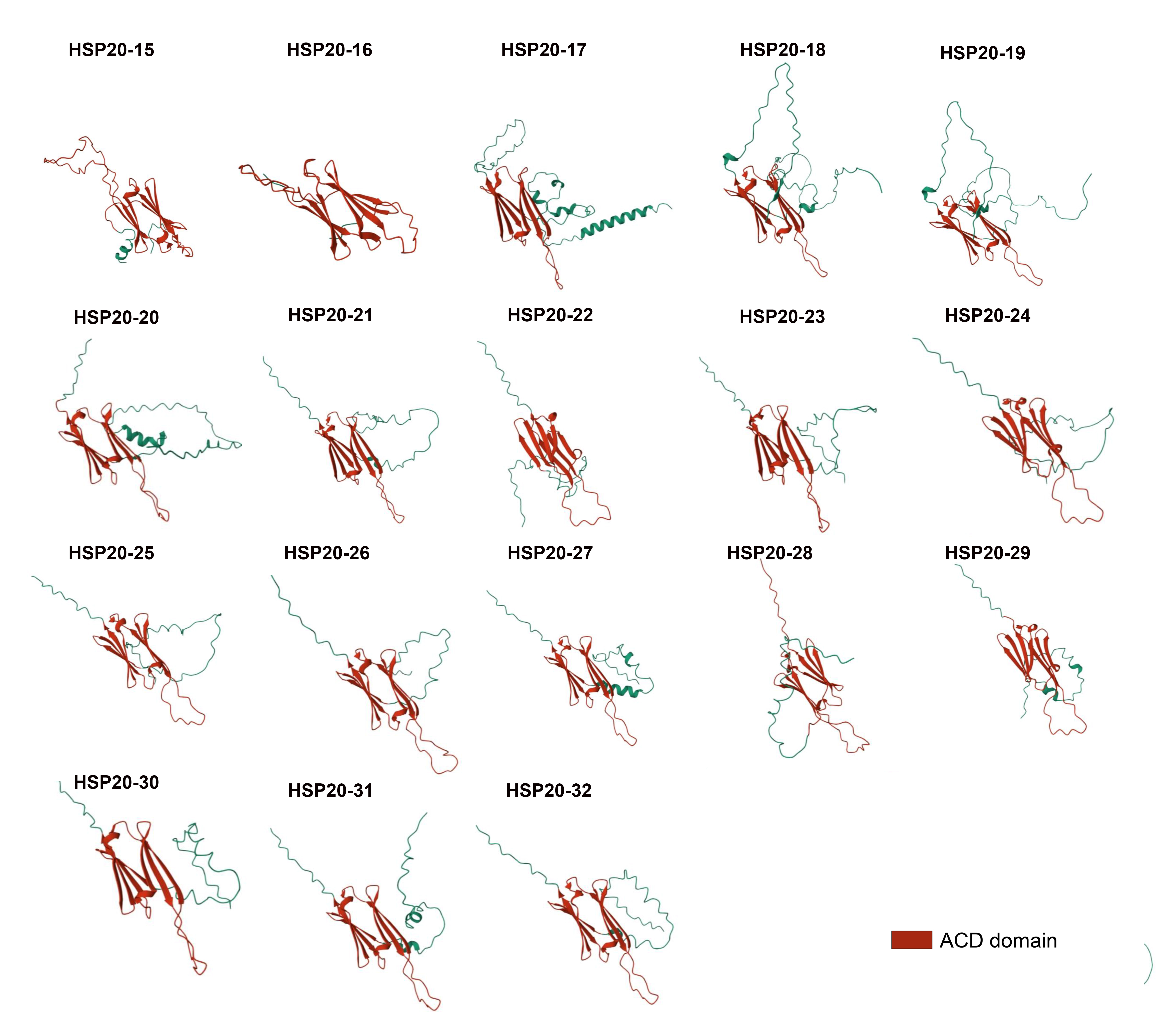

Supplement: Supplementary file 1 [file ijms-25-11550-s001.zip › FigureS3.jpg]

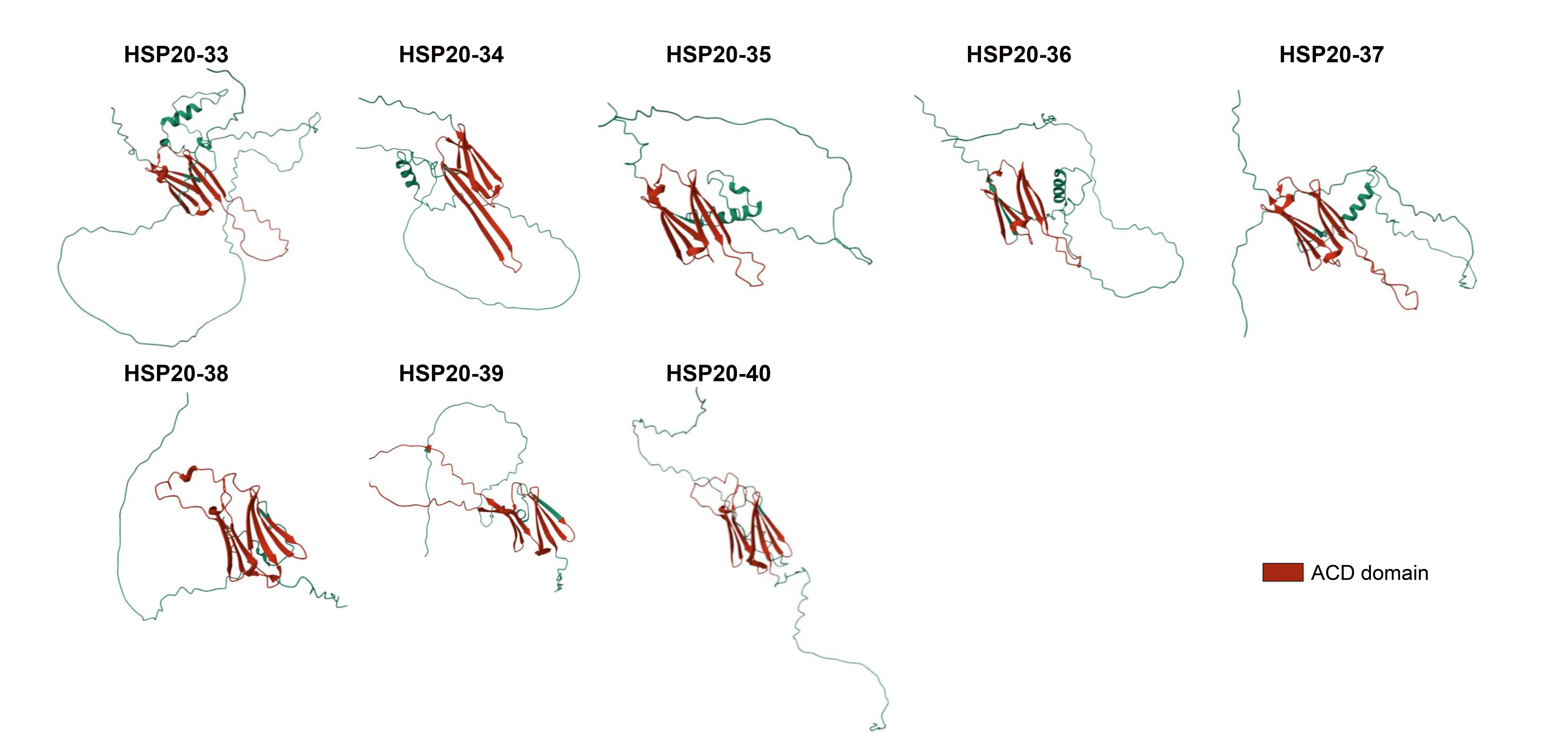

Supplement: Supplementary file 1 [file ijms-25-11550-s001.zip › FigureS4.jpg]

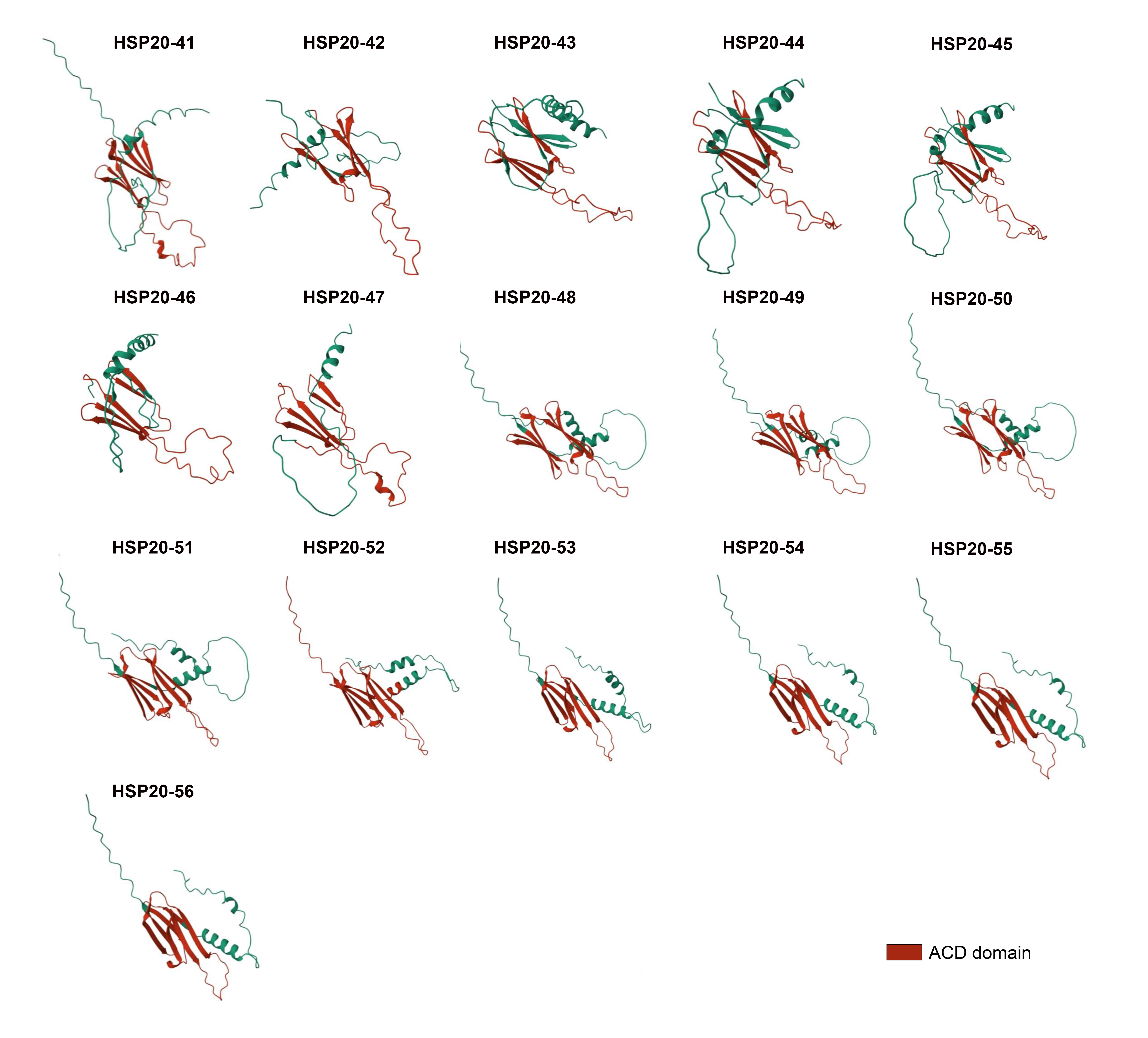

Supplement: Supplementary file 1 [file ijms-25-11550-s001.zip › FigureS5.jpg]

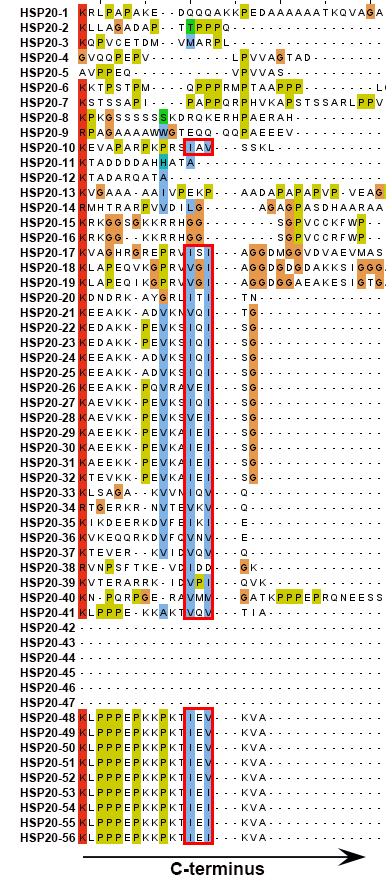

Supplement: Supplementary file 1 [file ijms-25-11550-s001.zip › FigureS6.jpg]

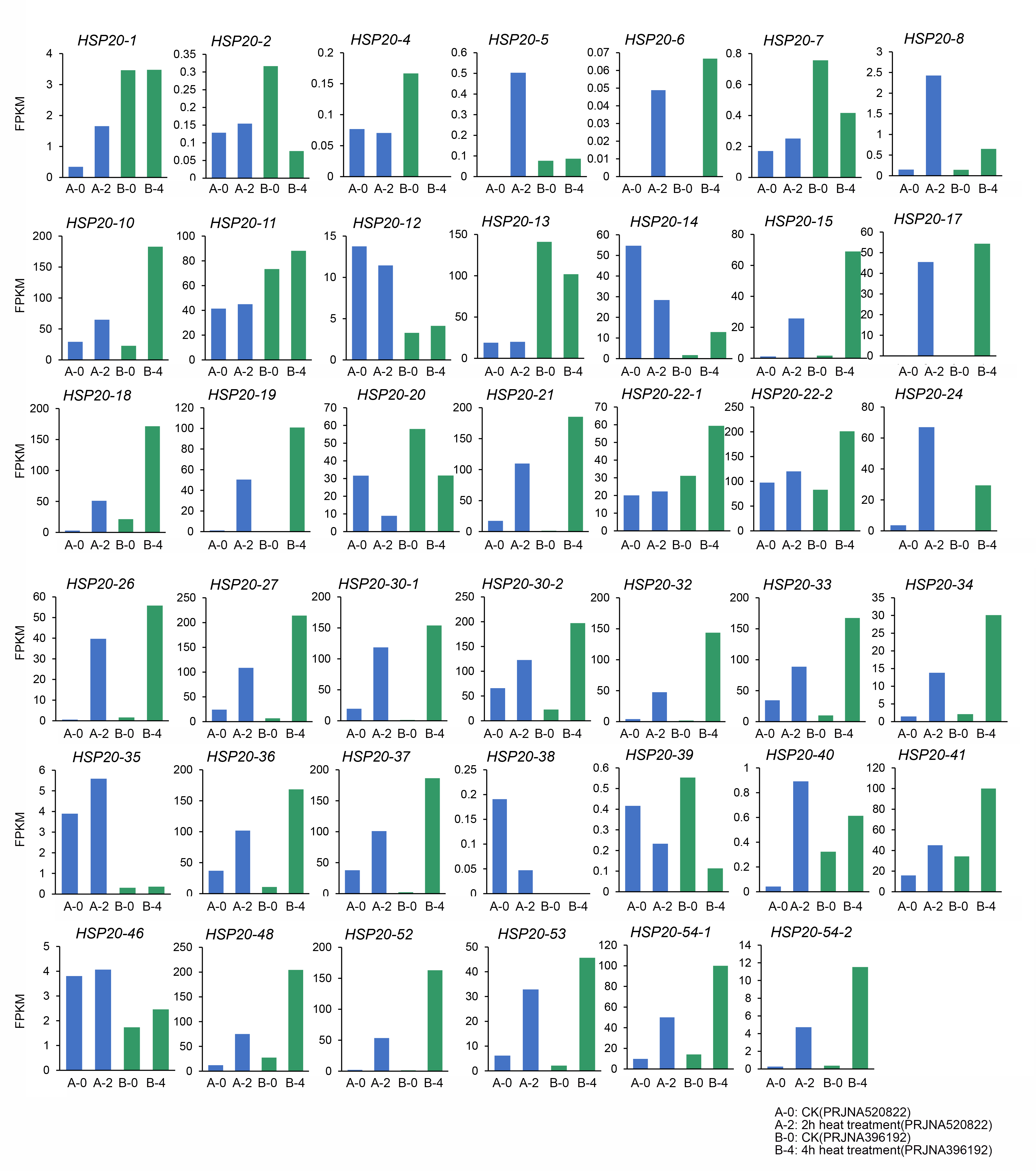

Supplement: Supplementary file 1 [file ijms-25-11550-s001.zip › FigureS7.jpg]

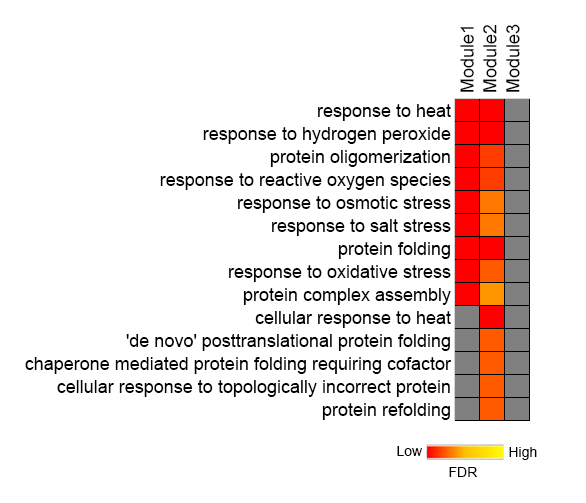

Supplement: Supplementary file 1 [file ijms-25-11550-s001.zip › FigureS8.jpg]
